# Application study of infrared free-electron lasers towards the development of amyloidosis therapy

**DOI:** 10.1107/S1600577522007330

**Published:** 2022-08-12

**Authors:** Mikiko Jindo, Kazuhiro Nakamura, Hisashi Okumura, Koichi Tsukiyama, Takayasu Kawasaki

**Affiliations:** a Department of Chemistry, Faculty of Science Division I, Tokyo University of Science, 1–3 Kagurazaka, Tokyo 184-8501, Japan; b Department of Laboratory Sciences, Gunma University, Graduate School of Health Sciences, 3-39-22, Showa-machi, Maebashi, Gunma 371-8511, Japan; c Exploratory Research Center on Life and Living Systems (ExCELLS), National Institutes of Natural Sciences, Okazaki, Aichi 444-8787, Japan; d Institute for Molecular Science, National Institutes of Natural Sciences, Okazaki, Aichi 444-8787, Japan; e Department of Structural Molecular Science, SOKENDAI (The Graduate University for Advanced Studies), Okazaki, Aichi 444-8787, Japan; f IR Free Electron Laser Research Center, Research Institute for Science and Technology, Organization for Research Advancement, Tokyo University of Science, 2641 Yamazaki, Noda, Chiba 278-8510, Japan; g Accelerator Laboratory, High Energy Accelerator Research Organization, 1-1 Oho, Tsukuba, Ibaraki 305-0801, Japan; RIKEN SPring-8 Center, Japan

**Keywords:** infrared free-electron laser, amyloidosis, amyloid fibril, β2-microglobulin

## Abstract

Amyloidosis is known to be caused by the deposition of amyloid fibrils into various biological tissues; effective treatments for the disease are little established today. An infrared free-electron laser (IR-FEL) is an accelerator-based picosecond-pulse laser having tunable infrared wavelengths. In the current study, the irradiation effect of an IR-FEL was tested on an 11-residue peptide (NFLNCYVSGFH) fibril from β2-microglobulin (β2M) with the aim of applying IR-FELs to amyloidosis therapy. Infrared microspectroscopy (IRM) and scanning electron microscopy showed that a fibril of β2M peptide was clearly dissociated by IR-FEL at 6.1 µm (amide I) accompanied by a decrease of the β-sheet and an increase of the α-helix. No dissociative process was recognized at 6.5 µm (amide II) as well as at 5.0 µm (non-specific wavelength). Equilibrium molecular dynamics simulations indicated that the α-helix can exist stably and the probability of forming interchain hydrogen bonds associated with the internal asparagine residue (N4) is notably reduced compared with other amino acids after the β-sheet is dissociated by amide I specific irradiation. This result implies that N4 plays a key role for recombination of hydrogen bonds in the dissociation of the β2M fibril. In addition, the β-sheet was disrupted at temperatures higher than 340 K while the α-helix did not appear even though the fibril was heated up to 363 K as revealed by IRM. The current study gives solid evidence for the laser-mediated conversion from β-sheet to α-helix in amyloid fibrils at the molecular level.

## Abstract 2

An infrared free-electron laser was applied to dissociate amyloid fibrils of a peptide as a goal of the development of a novel therapeutic strategy for amyloidosis. Solid evidence for β-sheet to α-helix conversion in the peptide fibril by vibrational excitation of amide bonds was obtained by both experiment and molecular dynamics simulation.

## 1. Introduction

Protein structures are generally constructed by gathering many peptide segments such as α-helix and β-sheets, and these are thermodynamically stable under physiological conditions. However, if an amino acid mutation occurs or stress factors such as hydrogen ion concentration and temperature are changed in the crowded milieu, highly hydro­phobic and β-sheet-dominant structures can be produced (Blokhuis *et al.*, 2013 ▸; Jin *et al.*, 2019 ▸; Simpson *et al.*, 2020 ▸). One such rigid protein aggregate is an amyloid fibril, which is a causal factor for serious amyloidosis such as Alzheimer’s disease (Guo & Xu, 2008 ▸; Marijan *et al.*, 2019 ▸; Qiang *et al.*, 2017 ▸). Although several pharmaceutical drugs such as antibodies and small molecules have been developed as therapeutic agents, effective treatments for amyloidosis are not yet established today (Khan *et al.*, 2020 ▸; Kushwah *et al.*, 2020 ▸; Yamamoto & Yokochi, 2019 ▸). An amyloid fibril is usually composed of a string assembly in which the diameter of one string ranges from 1 to 10 nm and its length is several micrometres. It has long been known that more than 30 kinds of proteins form an amyloid-like fibril structure, and interestingly those amyloid fibrils possess a common cross-β-sheet stacking conformation although the amino acids sequences are different (Benson *et al.*, 2018 ▸). In principle, it can be expected that degradation of the fibrous structure can lead to a blocking of the progression of the disease, which should become an efficient therapeutic strategy. However, the rigid β-sheet structure is formed by many intermolecular hydrogen bonds (Tsemekhman *et al.*, 2007 ▸; Wei *et al.*, 2016 ▸; Zhai *et al.*, 2019 ▸), and it is difficult to dissociate the aggregate under physiological conditions unless denaturants are used (Deckert-Gaudig & Deckert, 2016 ▸; Shen & Murphy, 1995 ▸).

Infrared free-electron lasers (IR-FELs) are accelerator-based femtosecond- to picosecond-pulse lasers and are generated by the strong interaction of the accelerated electron beam with synchrotron radiation in a periodic magnetic field, the so-called undulator (Glotin *et al.*, 1993 ▸; Grosse, 2002 ▸; Knippels *et al.*, 1998 ▸). A remarkable feature of the IR-FEL is that the oscillation wavelengths are tunable from the terahertz region to near-infrared wavelengths (3–300 µm), and there are many wavelengths that are resonant with various functional groups of organic compounds in the mid-infrared region (5–20 µm). IR-FELs can activate vibrational modes associated with specific chemical bonds selectively and induce remarkable structural changes on the various compounds, which is barely achievable by simple heating. There are several IR-FEL oscillation facilities around the world, and many application studies are performed in various research fields such as physical chemistry (Andersson *et al.*, 2020 ▸; Elferink *et al.*, 2018 ▸; Munshi *et al.*, 2019 ▸), material science (Kawasaki *et al.*, 2021 ▸; Kehr *et al.*, 2011 ▸; Shevchenko *et al.*, 2019 ▸) and biomedical sciences (Halliwell *et al.*, 2017 ▸; Kawasaki *et al.*, 2016*a* ▸; Peavy *et al.*, 1999 ▸; Wells *et al.*, 2005 ▸; Wilmink *et al.*, 2008 ▸; Xiao *et al.*, 2008 ▸). We have applied an IR-FEL to irradiate various types of proteins *in vitro*, and discovered that tuning the laser beam to 6.1–6.2 µm, which corresponds to the stretching vibrational mode of amide carbonyl bonds (νC=O, amide I) (Kawasaki *et al.*, 2012 ▸, 2014 ▸, 2016*b* ▸; 2018 ▸, 2020 ▸; Okumura *et al.*, 2021 ▸), and tuning to the terahertz frequency (3.5–4.5 THz, 70–80 µm) (Kawasaki *et al.*, 2019*a* ▸) can efficiently dissociate fibrous peptide structures. In most cases, the proportion of β-sheets is decreased and that of α-helices is increased by these irradiations. An underlying mechanism of amyloid fibril dissociation by the IR-FEL can be proposed – that a multi-photon absorption at the amide bonds can break the hydrogen-bond network in the fibrous structure and produce non-fibrous conformations. However, the dissociation processes vary with the peptide due to the difference of amino acid sequences: not only the α-helix but also the other conformations such as β-turns and random coils are increased in some types of amyloid fibrils from Aβ42, GNNQQNY, insulin and lysozyme (Kawasaki *et al.*, 2012 ▸, 2014 ▸, 2018 ▸, 2020 ▸; Okumura *et al.*, 2021 ▸). In addition, discussion of the fibril dissociation process at the amino acid sequence level has been described only in a study on GNNQQNY peptide (Kawasaki *et al.*, 2020 ▸). Therefore, further studies of the conformational change of the amyloid fibril should be amassed to develop an IR-FEL mediated therapeutic approach for amyloidosis.

In the current study, we tested the irradiation effect of the IR-FEL on the disaggregation of the amyloid fibril by using β2-microglobulin (β2M) peptide as a new model sample. β2M is closely associated with dialysis-related amyloidosis (Scarpioni *et al.*, 2016 ▸), and the internal 11-residue peptide (NFLNCYVSGFH) easily forms a fibrous aggregate under physiological conditions (Nishino *et al.*, 2005 ▸). A fibril of β2M peptide was irradiated by the IR-FEL, and conformational changes of the peptide fragment were analysed by both microscopic observations and molecular dynamics simulation.

## 2. Experimental

### 2.1. Materials

β2-Microglobulin peptide fragment (NFLNCYVSGFH, 85% purity) was purchased from PH Japan Co. Ltd (Hiroshima, Japan). Di­methyl sulfoxide (DMSO) was obtained from Nacalai Tesque, Inc. (Kyoto, Japan). Phosphate buffer saline (PBS) and sodium chloride were purchased from FUJIFILM Wako Pure Chemical Corporation (Osaka, Japan). Congo-red was obtained from Sigma-Aldrich (St Louis, MO, USA).

### 2.2. Fibrillation of peptide

Freeze-dried β2-microglobulin peptide was dissolved in DMSO to 40 mg ml^−1^, and the stock solution was diluted with 0.1 *M* PBS containing 0.1 *M* sodium chloride to 2.0 mg ml^−1^ and incubated at room temperature for four days.

### 2.3. IR-FEL irradiation

The IR-FEL was oscillated briefly as follows: an electron beam generated by a high radio-frequency (RF) electron gun (2856 MHz) is accelerated by a linear accelerator and introduced to an undulator (a periodic magnetic field). The maximum acceleration energy is 40 MeV, and the electron beam is forced to oscillate the synchrotron radiation in the undulator. The synchrotron radiation is amplified between two resonant mirrors to interact with the successive electron beam, which can produce the IR-FEL. A fraction of the laser pulse is taken out through a coupling hole in the upstream mirror and led to a laboratory. The IR-FEL at Tokyo University of Science, Japan, covers mid-infrared wavelengths from 5 to 10 µm and is composed of two types of pulses, a macro-pulse and a micro-pulse. The macro-pulse has a duration of 2 µs with a repetition rate of 5 Hz, and the micro-pulse has a duration of 2 ps and an interval of two consecutive micro-pulses of 350 ps. A portion of the peptide fibril suspension (10 µL) was added on a metal-coated plate for infrared microspectroscopy analysis or a glass slide base for scanning-electron microscopy observation and dried under atmosphere at room temperature. Laser beams of the IR-FEL tuned to various wavelengths were guided onto the sample surface using a BaF_2_ lens, and the beam diameter was controlled by changing the height of the sample stage to set the fluence from 10 to 50 mJ cm^−2^.

### 2.4. Infrared microspectroscopy (IRM)

We used an IRM-7000 infrared microscope combined with an FT/IR-6100 series spectroscope (Jasco Co.). The surface of the sample dried on the metal-coated plate was observed using a 16× Cassegrain lens, and the IR spectra were recorded in 32 scans in reflection mode from 600 to 4000 cm^−1^. The aperture size was set to 100 µm × 100 µm for every measurement.

### 2.5. Peptide conformation analysis

For estimation of the proportions of the peptide conformations, *IR-SSE* analytical software (Jasco Co.) was used (Sarver & Krueger, 1991 ▸). The amide I band observed between 1600 and 1700 cm^−1^ can be divided into four bands: α-helix (1650–55 cm^−1^), β-sheet (1625–40 cm^−1^), turn (1655–75 cm^−1^) and non-ordered conformation (1645–50 cm^−1^), and the proportion of the conformation was obtained from the ratio of the peak intensity of each conformation to that of the whole amide I band. Those data were statistically analysed using several peptide samples before or after the irradiations.

### 2.6. Congo-red staining

The reagent was dissolved in PBS to 0.2 m*M* concentration, and a portion of the solution (10 µL) was added to the peptide solution (2.0 mg ml^−1^) at room temperature. The mixture was incubated for several minutes on a glass slide. The green–yellow birefringence was observed using a polarized light microscope (MVX10, Olympus, Tokyo, Japan).

### 2.7. Scanning-electron microscopy (SEM)

We used an FE-SEM Supra40 scanning electron microscope (Carl Zeiss) for SEM imaging. A dried sample of the peptide fibril on a glass slide base was fixed on a sample holder using conductive copper tape. The surface of the sample was observed using an acceleration voltage of 3.0 or 5.0 kV.

### 2.8. Equilibrium molecular dynamics simulation (EMDS)

We performed equilibrium molecular dynamics simulations of β2-microglobulin (β2M) peptide using *GROMACS* (version 2019.6). The four β-sheet bundle of β2M was extracted from the 21–31 fragment of the NMR structure (PDB code: 2e8d) (Iwata *et al.*, 2006 ▸). The α-helix was produced by restraining the dihedral angle (φ = −60°, ψ = −45°) of the main chain. The time step was set to 2 fs. AMBER96 and TIP3P were used as the force fields of the protein and water, respectively. Water molecules were deployed in a solvent box that was equilibrated under 300 K, 1 atm, and sodium ions and chloride ions were replaced with water molecules to set the ionic strength to 0.1 *M*, similar to the experimental conditions. In this step, the total electric charge was set to zero. The energy minimizations of these systems were performed for 1000 steps. The equilibration runs were performed for 50000 steps while keeping constant the number of particles, temperature and volume. The production runs were then performed for 50 ns while keeping constant the number of particles, temperature and pressure. The Particle Mesh Ewald method (Essmann *et al.*, 1995 ▸) and Berendsen method (Berendsen *et al.*, 1984 ▸) were used for electrostatic interaction and temperature control, respectively. The Parrinello–Rahman method (Parrinello & Rahman, 1980 ▸) was used for pressure control. As for the stability of the protein secondary structures and hydrogen bond analysis, *DSSP* (version 2.0.4) software and *VMD* (version 1.9.3) visualization software, respectively, were used.

## 3. Results

### 3.1. Conformational analysis by IRM

**Figure 1:**
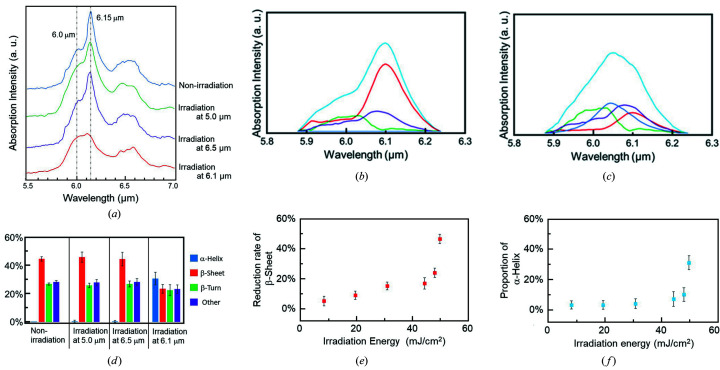
Experimental analysis. (*a*) Infrared spectra of β2M peptide fibril in the amide I and II regions. Blue: non-irradiation peptide; green: peptide after irradiation at 5.0 µm; violet: peptide after irradiation at 6.5 µm; red: peptide after irradiation at 6.1 µm. (*b*) Deconvolution spectra before irradiation. Blue: α-helix; red: β-sheet; green: β-turn; violet: other conformation; light blue: total spectra. (*c*) Deconvolution spectra after irradiation at 6.1 µm. (*d*) Protein secondary conformation analysis before (non-irradiation) and after irradiations at 5.0, 6.5 and 6.1 µm. Blue bar: α-helix; red bar: β-sheet; green bar: β-turn; violet bar: other conformation. (*e*) Effect of laser energy fluence on the reduction of the β-sheet of β2M peptide. (*f*) Effect of laser energy fluence on the proportion of the α-helix of β2M peptide.

We selected three wavelengths (6.1, 6.5 and 5.0 µm) for the irradiation: 6.1 µm corresponds to amide I (νC=O), 6.5 µm corresponds to amide II (δN—H), while the sample shows little absorption at 5.0 µm. In the FT-IR spectra of β2M fibril [Fig. 1 ▸(*a*)], the main peak of the amide I band was observed at 6.15 µm (1624 cm^−1^), corresponding to the β-sheet (blue, non-irradiation), and that of the amide bonds associating with the other conformations is observed as a broad peak at about 6.0 µm (1667 cm^−1^) (Komatsu *et al.*, 2007 ▸). The beam diameter was adjusted to about 0.2 cm where the energy fluence reached 50 mJ cm^−2^ at maximum. After the irradiation was tuned to 6.1 µm (red), the peak intensity at 6.15 µm was clearly decreased compared with those tuned to 6.5 µm (violet) and 5.0 µm (green). The amide I band can be deconvoluted to four bands corresponding to the α-helix (blue), β-sheet (red), β-turn (green) and other conformations (violet) as shown in Fig. 1 ▸(*b*). Those deconvolution spectra showed clear differences of the four conformations in spectral shapes and widths. The β-sheet that has a peak at 6.1 µm was the main band, and the α-helix was only slightly observed before irradiation. On the contrary, the peak intensity of the β-sheet was decreased and the α-helix band appeared after the irradiation at 6.1 µm [Fig. 1 ▸(*c*)]. As shown by the protein secondary structure analysis [Fig. 1 ▸(*d*)], the β-sheet proportion (red bar) is about 45% and that of the α-helix (blue bar) is only slightly recognized in the non-irradiation fibril. Irradiation at neither 5.0 µm nor 6.5 µm changed those conformations. On the other hand, the irradiation tuned to 6.1 µm reduced the proportion of the β-sheet to near 25% and increased that of the α-helix to 30%. Both the β-turn (green bar) and other conformations (violet bar) were slightly decreased.

The effect of the energy fluence of the IR-FEL on the reduction rate (r.r.) of β-sheet conformation was examined [Fig. 1 ▸(*e*)]. The r.r. value was calculated using the following equation,

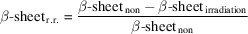

where β-sheet_r.r._ is the reduction rate of β-sheet conformation, β-sheet_non_ is the β-sheet proportion before irradiation, and β-sheet_irradiation_ is the β-sheet proportion after irradiation.

When the energy density of the laser beam was increased from 10 to 20 mJ cm^−2^, the r.r. value of the β-sheet did not change much, but it increased remarkably when the energy density was more than 40 mJ cm^−2^. The α-helix content was increased at 40–50 mJ cm^−2^ accordingly [Fig. 1 ▸(*f*)].

**Figure 2:**
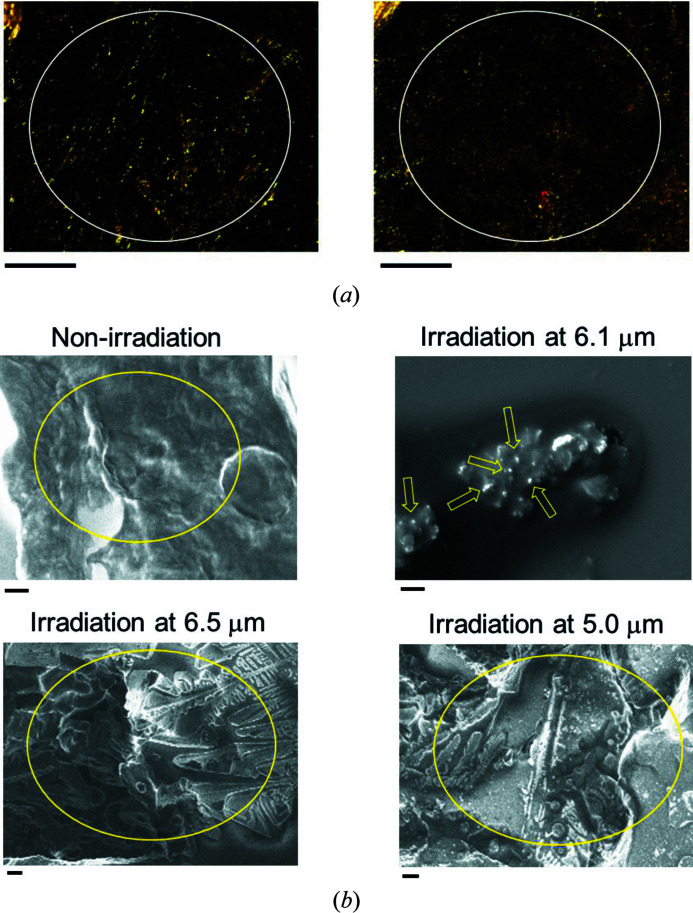
Morphological observation. (*a*) Congo-red staining before (left) and after (right) irradiation at 6.1 µm. Black bar: 100 µm. (*b*) SEM observation. Upper left: β2M peptide fibril before irradiation; upper right: β2M peptide fibril after irradiation at 6.1 µm; bottom left: β2M peptide fibril after irradiation at 6.5 µm; bottom right: β2M peptide fibril after irradiation at 5.0 µm. Black bar: 1 µm.

In addition, we observed the morphological change of the β2M fibril by using Congo-red staining and SEM (Fig. 2 ▸). It is well known that Congo-red binds to β-sheet stacks of amyloid fibrils and emits green–yellow birefringent light (Jagusiak *et al.*, 2019 ▸). Before the IR-FEL irradiation [left, Fig. 2 ▸(*a*)], a brilliant light can be observed which indicates the presence of fibrils (white circle). On the contrary, the strength of the light seemed to be weakened after the irradiation at 6.1 µm (right), which may indicate that the fibril state can be overall dissociated by the irradiation. In the SEM images [Fig. 2 ▸(*b*)], a soft-cloth-like fibril of β2M was observed before the irradiation (upper left, yellow circle). Those strings had clearly disappeared after IR-FEL irradiation tuned to 6.1 µm (upper right), and it can be estimated that several small particles whose diameters will be about 100–200 nm (yellow arrows) should be non-fibril assemblies (Kawasaki *et al.*, 2018 ▸). Those structural changes seem to indicate that the fibril was not blown away but transformed into the non-fibril state by the irradiation. On the other hand, many solid fibrils survived after both irradiations at 6.5 µm (bottom left) and 5.0 µm (bottom right) as indicated by the yellow circles. These morphological observations also showed amide I specific dissociation of peptide fibrils.

### 3.2. EMDS study

#### 3.2.1. Stabilities of the α-helix and β-sheet

**Figure 3:**
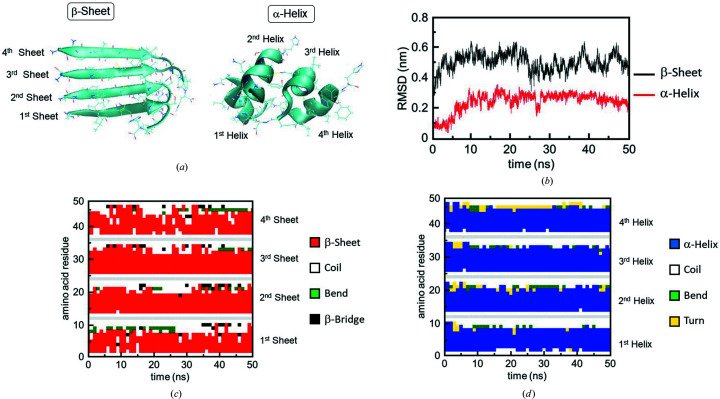
Equilibrium molecular dynamics simulation. (*a*) Initial β-sheet (left) and α-helix (right) models of β2M peptide. The four β-sheets were extracted from the 21–31 fragment, and the α-helix was produced by restricting the dihedral angle of the main chain. (*b*) RMSD values (nm) of α-helix (red) and β-sheet (black). (*c*) Time evolution of β-sheets in four bundles of β2M peptide. Red: β-sheet; white: random coil; green: bend; black: β-bridge. (*d*) Time evolution of α-helices in four bundles of β2M peptide. Blue: α-helix; white: random coil; green: bend; yellow: turn.

Initial conformations for the α-helix and β-sheet were obtained under the restriction of the dihedral angles of the amide bonds [Fig. 3 ▸(*a*)]. An individual number was given to each peptide for convenience. Molecular dynamics simulations of the α-helix and β-sheet were performed for 50 ns, and root-mean-square-deviation (RMSD) values are plotted against the simulation time in Fig. 3 ▸(*b*). The converged value of the β-sheet is 0.5, and that of the α-helix is 0.25, and the fluctuation of the α-helix was smaller than that of the β-sheet from 10 to 50 ns. Next, the time evolutions of the β-sheet and α-helix in β2M peptide were examined [Figs. 3 ▸(*c*) and 3(*d*)]. In the case of the β-sheet (red), several internal amino acids can be seen showing random coils (white) at the 1st and 4th sheets. On the contrary, in the case of the α-helix (blue), other conformations than the α-helix are rare for all helices during the simulation period. These parameters including RMSD values suggest that the α-helix can exist stably once the β-sheet is disrupted by the IR-FEL irradiation tuned to the amide I band.

#### 3.2.2. Analysis of hydrogen bonds

**Figure 4:**
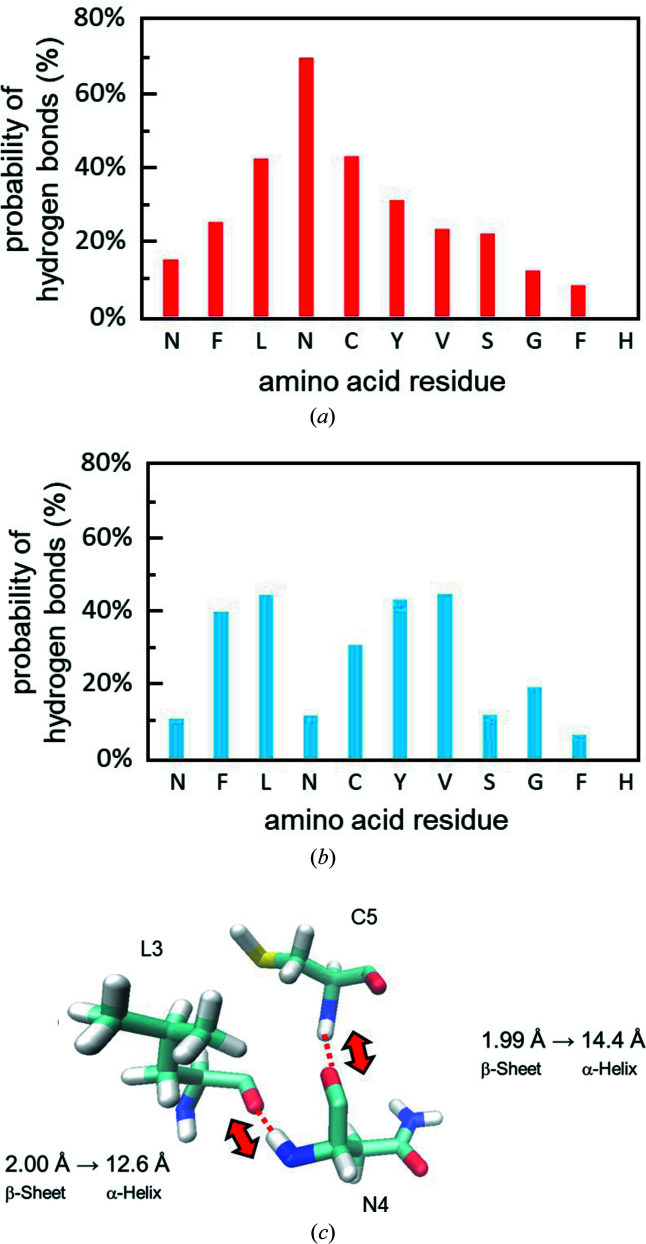
Hydrogen bonds analysis and 3D stick model focusing for N4. (*a*) Probability of hydrogen bonds in the β-sheet. Horizontal axis: amino acid sequence of the 21–31 fragment of β2M peptide. (*b*) Probability of hydrogen bonds in the α-helix. The horizontal axis is the same as in (*a*). (*c*) Change of distances of the hydrogen bonds between N4 and L3 or C5 in the conversion from β-sheet into α-helix. Light blue stick: carbon; red: oxygen; blue: nitro­gen; white: hydrogen.

To investigate how a β-sheet can be converted into an α-helix after IR-FEL irradiation, the hydrogen bonds forming between the main peptide chains were analysed (Fig. 4 ▸). In the β-sheet conformation, the probability of hydrogen bond formation was comparatively high at the internal three amino acids (L3, N4 and C5) [Fig. 4 ▸(*a*)], whereas it was significant at four amino acids (F2, L3, Y6 and V7) in the α-helix [Fig. 4 ▸(*b*)]. Remarkably, the probability of hydrogen bond formation was reduced from 69.6% in the β-sheet to 11.7% in the α-helix at the internal N4. Hydrogen bond patterns associated with N4 are shown in Fig. 4 ▸(*c*). The 3D stick model showed that the distances of the hydrogen bond (C=O⋯H—N) between N4 and L3 or C5 in the β-sheet were 2.00 Å and 1.99 Å, respectively. The probability of hydrogen bond formation of the β-sheet associated with N4 (69.6%) was divided into two patterns: 32.2% (hydrogen bond between N—H of N4 and C=O of L3) and 37.4% (hydrogen bond between C=O of N4 and N—H of C5). On the contrary, those distances were elongated to 12.6 Å and 14.4 Å in the α-helix. These simulation results mean that IR-FEL irradiation can dissociate the hydrogen bonds between the main chains associated with the internal N4 and suggest that N4 plays an important role in the conversion from β-sheet to α-helix.

#### 3.2.3. Effect of steady heating on β-sheet conformation

**Figure 5:**
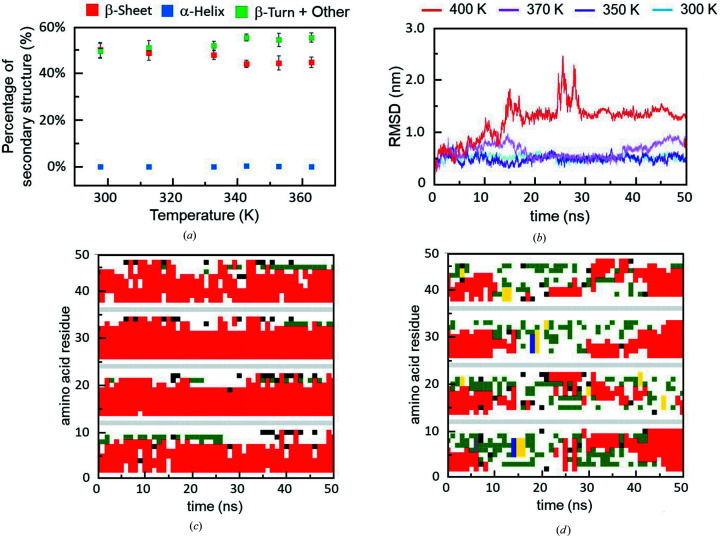
Comparison with steady heating. (*a*) Secondary conformation analysis using infrared microspectroscopy (IRM). Heating was performed for 30 min at each temperature (six points) from 298 to 363 K. Red: β-sheet; blue: α-helix; green: β-turn and other conformation. (*b*) Time evolution of RMSD (nm) at different temperatures. Light blue: 300 K; violet: 350 K; magenta: 370 K; red: 400 K. (*c*) Simulation of the stability of the β-sheet in four bundles of β2M peptide at 300 K. Red: β-sheet; white: random coil; green: bend; black: β-bridge. (*d*) Simulation of the stability of the β-sheet in four bundles of β2M peptide at 400 K. Colour coding is the same as in (*c*).

We compared the effect of the IR-FEL irradiation with that by simple steady heating (Fig. 5 ▸). The conformational change of the β2M peptide fibril was analysed during heating from 298 to 363 K in a similar way using IRM as shown in Fig. 1 ▸(*d*), and the proportion of the β-sheet (red) was slightly decreased at temperatures higher than 340 K accompanied by increases of β-turns and the other conformations (green), while α-helix (blue) did not appear at any temperatures [Fig. 5 ▸(*a*)]. This result showed that the increase of the α-helix that was observed for the IR-FEL irradiation tuned to 6.1 µm is not due to simple thermal effects and implies that selective vibrational excitation of amide bonds should be required for the β-sheet to α-helix conversion.

The RMSD evolution of the β-sheet during the heating is shown in Fig. 5 ▸(*b*). At 300 K (light blue) or 350 K (violet), the RMSD value was around 0.5 nm, and the fluctuation is small. On the contrary, the fluctuation is slightly elevated at 370 K (magenta), and the RMSD was more than 1.0 nm at 400 K (red). The time evolutions of the secondary conformations at 300 K and at 400 K are shown in Figs. 5 ▸(*c*) and 5(*d*). Although the β-sheet is dominant at 300 K [Fig. 5 ▸(*c*)], the other conformations and random coils are increased and the β-sheet is reduced for most amino acid residues at 400 K [Fig. 5 ▸(*d*)]. These simulation results indicate that the β-sheets are unstable at temperatures higher than 350 K, which is consistent with the experimental result that was obtained by the IRM analysis [Fig. 5 ▸(*a*)].

## 4. Discussion

We have found that various types of amyloid fibrils can be dissociated by IR-FEL irradiation tuned to the amide I band, and the dissociation pattern varies with the molecular types and amino acid sequences of amyloid peptides as follows: not only α-helix but also the other conformations such as β-turn and random coils are obviously increased by the irradiation in the cases of Aβ (42 residues) (Kawasaki *et al.*, 2018 ▸; Okumura *et al.*, 2021 ▸), GNNQQNY (7 residues) (Kawasaki *et al.*, 2020 ▸), insulin (51 residues) (Kawasaki *et al.*, 2014 ▸) and lysozyme (129 residues) (Kawasaki *et al.*, 2012 ▸). On the contrary, the other conformations are little changed in the cases of polyglutamine (69 residues) (Kawasaki *et al.*, 2016*b* ▸) and DFNKF (5 residues) (Kawasaki *et al.*, 2019*a* ▸), and are rather decreased in the case of β2-microglobulin peptide (11 residues) in the present study. As shown in these examples, it is difficult to explain the variety of conformational changes by IR-FEL irradiation on the grounds of the differences of the amino acid sequences. Nonetheless, the decrease of β-sheets accompanying the increase of α-helices is a common phenomenon in all peptides. This implies that targeting the amide I vibrational excitation with the IR-FEL should be a versatile and robust approach for the dissociation of the various types of amyloid fibrils. As shown by the non-equilibrium molecular dynamics (NEMD) simulation of Aβ peptide fibril which was performed by Okumura *et al.* (2021 ▸), the resonant wavelength at the amide I band is also important for the reduction not of the α-helix but the β-sheet, and it can be suggested that the vibrational excitation at lower wavenumbers (1620–1640 cm^−1^) that corresponds to νC=O of the β-sheet is a key point for the dissociation of the fibrous conformations.

As for the probability of hydrogen bond formation, the NEMD simulation conducted by Dr Nguyen and collaborators showed that the number of hydrogen bonds in contact with the internal glutamine residue in GNNQQNY peptide was decreased by the IR-FEL irradiation (Kawasaki *et al.*, 2020 ▸). Namely, the β-sheet of the seven-residue peptide can be reduced by the dissociation of the intermolecular hydrogen bonds associated with the internal amino acids. The current study using 11-residue peptide also implies that the probability of hydrogen bond formation of the internal amino acid (N4) is substantially reduced with considerable decrease of the β-sheet structure. Putting such experimental and numerical results together, it is believed that hydrogen bond cleavage of the internal amino acid is a key point for the β-sheet to α-helix conversion in the peptide fibril by amide I selective irradiation.

In dialysis patients, β2-microglobulin accumulates and aggregates within the carpal tunnel, and the fibrous aggregate oppresses the median nerve (Scarpioni *et al.*, 2016 ▸; Wipperman & Goerl, 2016 ▸), which is the pathogenesis of carpal tunnel syndrome. It is the most common cause of peripheral entrapment neuropathy (Ghasemi-rad *et al.*, 2014 ▸). Opening of the carpal tunnel, the general operative method, might be applied for patients with mild symptoms. However, additional operations such as synovectomy, flexor tendon reduction and superficial flexor digital tendon extraction are likely needed for patients with severe symptoms due to long-term dialysis. Therefore, novel non-operative therapeutic strategies for carpal tunnel syndrome provide alternative choices of therapies to the patients. Removing the β2M fibril from the deposited tissues is theoretically effective as a therapeutic approach. Therefore, a permeable dialysis membrane has been traditionally used for the hemodialysis to treat the disease (Koch, 1992 ▸). We suggest that the amide I specific vibrational excitation using IR-FEL might be an alternative approach for carpal tunnel syndrome therapy. However, we have to clarify whether the laser irradiation impairs non-aggregated normal proteins. One hypothesis was proposed by our research group using hen egg-white lysozyme as a native protein model (Kawasaki *et al.*, 2019*b* ▸). In that study, we irradiated the lysozyme that is not fibrillated and found that the protein conformation was little changed although the enzymatic activity was slightly reduced by the amide I targeting excitation. Therefore, it can be expected that the IR-FEL irradiation can dissociate the fibrous structure of amyloid peptides without large conformational change of the other normal protein. Nonetheless, it is not yet clear how the IR-FEL irradiation can affect the internal tissues of the mammalian body. To clarify the effects of intense infrared radiation on physiological functions of the biological tissues is an important subject towards the development of the treatment of amyloidosis including carpal tunnel syndrome, and we would like to try to irradiate the tissues extracted from patients with carpal tunnel syndrome in the future.

## 5. Conclusion

We tested the irradiation effect of an IR-FEL on an amyloid fibril of β2-microgloblin 11-residue peptide. IRM analysis showed that the β-sheet was decreased and the α-helix was increased by the IR-FEL irradiation tuned to amide I (6.1 µm), and the conversion rate was gradually elevated dependent on the energy fluence up to 50 mJ cm^−2^. The dissociation of the fibril was also observed by Congo-red staining and scanning-electron microscopy, and the effect was hardly recognized at the irradiation tuned to amide II (6.5 µm) or a non-specific wavelength (5.0 µm). Equilibrium molecular dynamics simulation showed that the α-helix can be stably formed once the β-sheet is disrupted by IR-FEL irradiation from the RMSD plots and the time evolution of each conformation during 50 ns. Hydrogen bond analysis showed that the interchain hydrogen bonds associated with the internal asparagine residue (N4) were remarkably reduced compared with the other amino acids in the conversion from β-sheet to α-helix. In addition, IRM shows that the β-sheet can be destroyed without a noticeable increase of the α-helix under heating conditions up to 363 K. This proved that the conversion from β-sheet to α-helix induced by the IR-FEL is not due to simple thermal effects. The instability of the β-sheet was also evident from the RMSD and time evolution analyses by the EMDS. The IR-FEL irradiation can be proposed to be robust and universal for the destruction of amyloid fibrils *in vitro* and can be expected to be developed as a novel therapeutic technology for amyloidosis by examination of *in vivo* use in future.
